# Real-world efficacy of eculizumab in generalized myasthenia gravis patients with poor early response to efgartigimod: a prospective cohort study

**DOI:** 10.3389/fimmu.2026.1730742

**Published:** 2026-05-25

**Authors:** Xueting An, Lijie Zhu, Xiaoyu Huang, Zhouao Zhang, Yong Zhang, Chao Zhang

**Affiliations:** 1 Department of Neurology, Tianjin Neurological Institute, Tianjin Medical University General Hospital, Tianjin, China; 2 Department of Neurology, The Affiliated Hospital of Xuzhou Medical University, Xuzhou, Jiangsu, China

**Keywords:** eculizumab, generalized myasthenia gravis, poor early response to efgartigimod, prospective cohort study, sequential therapy

## Abstract

**Objective:**

The management of generalized myasthenia gravis (gMG) poses a therapeutic challenge. Efgartigimod, a neonatal Fc receptor (FcRn) inhibitor, has significant efficacy in patients with positive acetylcholine receptor (AChR) antibodies. However, approximately 30% of patients exhibit a poor early response. This study aimed to explore whether the complement C5 inhibitor, eculizumab, can bring clinical benefits to patients who have a poor early response to efgartigimod, thereby investigating potential sequential treatment strategies.

**Methods:**

We first conducted a prospective study to rigorously evaluate and longitudinally follow up 103 patients with gMG who received one treatment cycle of efgartigimod, ultimately enrolling 18 patients who demonstrated an inadequate response to the therapy. Then, these patients received eculizumab induction (900 mg weekly for four weeks) followed by maintenance dosing (1200 mg every two weeks or extended to once every three weeks). Clinical outcomes were assessed using MG-specific activities of daily living (MG-ADL) and quantitative myasthenia gravis (QMG) scores. Safety was evaluated through adverse event reporting.

**Results:**

Significant clinical improvements were observed during the induction phase, with mean MG-ADL scores demonstrating a reduction from 7.9 ± 3.9 before eculizumab to 2.6 ± 1.7 [-5.3 (95% CI: -7.6 to -3.0); P < 0.001], while QMG scores dropped from 14.1 ± 6.6 to 6.5 ± 3.2 [-7.4 (95% CI: -10.2 to -4.7); P < 0.001]. All patients achieved clinically meaningful improvement (≥2-point MG-ADL reduction), with four patients reaching minimal symptom expression (MSE, defined as an MG-ADL score ≤1-point) by week 4. Among the 12 patients who completed the 16-week follow-up, seven patients (58.3%) achieved minimal symptom expression. Glucocorticoid doses were successfully reduced to ≤5mg per day in seven patients. Two mild adverse events (urinary tract infection and limb edema) were reported.

**Conclusion:**

Eculizumab exhibited rapid and significant efficacy in patients with gMG who were poor early response to efgartigimod, and exhibited a favorable safety profile. These findings suggest that complement inhibition may serve as a potential sequential therapy when FcRn antagonist modulation failure.

## Introduction

Myasthenia gravis (MG) is a classical autoimmune disorder characterized by pathogenic autoantibodies directed against the post-synaptic membrane of the neuromuscular junction, with the most prevalent being acetylcholine receptor (AChR) antibodies (1). The typical clinical features of MG are fluctuating skeletal muscle fatigue and weakness that can be aggravated by activity (1). MG can be classified into ocular and generalized subtypes depending on the muscle groups involved, with the majority of patients demonstrating generalized manifestations characterized by weakness in the extraocular, bulbar, truncal, and limb muscles (2). Retrospective studies have identified generalized myasthenia gravis (gMG) as a factor that hinders patients from achieving minimal manifestation status or better (3, 4). Particularly in severe cases, patients with gMG may progress to a myasthenic crisis, which can result in life-threatening respiratory failure requiring intubation or non-invasive ventilation (5). Therefore, patients with gMG urgently need active and individualized treatment based on scientific evidence and established clinical guidelines.

Traditional treatment strategies for gMG follow a stepwise approach beginning with acetylcholinesterase inhibitors, corticosteroids, and conventional immunosuppressants, such as azathioprine, mycophenolate mofetil, and tacrolimus (2, 6). Intravenous immunoglobulin and plasma exchange are usually used for patients with impending crisis or myasthenic crisis (2, 6). Most patients experience mild to minimal symptoms or even achieve remission. However, 10%-20% of patients continue to exhibit moderate to severe weakness over the long term, despite treatment with at least two different high-dose immunosuppressive agents (7). These current challenges have ushered in a new era of biologic-based targeted therapy for the treatment of MG. Efgartigimod, a human IgG1 antibody Fc fragment that competitively inhibits the neonatal Fc receptor (FcRn), was approved in China in 2023 based on results from the phase 3 ADAPT trial, which demonstrated rapid reduction of immunoglobulin G (IgG) and clinical improvement in patients with generalized MG (8). However, approximately 30% of patients still demonstrated a suboptimal response to the first cycle of efgartigimod (8). Real-world evidence from the UK demonstrates that among patients with gMG treated with efgartigimod, 75% achieved a reduction of ≥2 points in the Myasthenia Gravis Activities of Daily Living (MG-ADL) score during the first treatment cycle. Nevertheless, 25% of patients did not reach the predefined treatment goal (9). To our knowledge, existing studies have primarily focused on the efficacy and safety of efgartigimod in patients with gMG, with no published reports addressing the optimal subsequent treatment strategies for patients who fail to respond to this therapy.

The complement system also plays a pivotal role in the pathogenesis of MG, especially in patients who are positive for AChR antibodies. Autoantibodies trigger the activation of the complement cascade, resulting in the formation of the membrane attack complex (MAC) and the subsequent disruption of postsynaptic membranes (10, 11). Eculizumab is a humanized monoclonal antibody that binds to the complement protein C5, thereby preventing the formation of the pro-inflammatory molecules C5a and the MAC (12). Additionally, it has received subsequent approval by the U.S. Food and Drug Administration for MG treatment. Real-world evidence from the United States demonstrates that eculizumab use in patients with gMG is associated with improved outcome measures, including reductions in MG-ADL and QMG scores, decreased reliance on concomitant therapies such as IVIg, prednisone, and pyridostigmine, as well as reduced frequency of myasthenia gravis (MG) crises and exacerbations and related hospitalizations (13). Furthermore, clinical benefits of eculizumab were confirmed in a 1-year post-marketing surveillance study in Japan, which showed sustained improvements in MG-ADL and QMG scores, a high responder rate, and consistent reduction in daily oral corticosteroid dosage (14).

Currently, there are limited data on subsequent therapeutic options for patients refractory to efgartigimod, and the therapeutic potential of complement inhibitors in those with inadequate responses to efgartigimod remains to be fully elucidated. Thus, this study aims to evaluate the efficacy and safety of eculizumab in gMG patients with poor early response to efgartigimod.

## Materials and methods

### Subjects

This study first conducted a prospective study to rigorously evaluate and longitudinally follow up 103 patients with gMG who received one treatment cycle of efgartigimod at the Affiliated Hospital of Xuzhou Medical University, ultimately enrolling 18 patients who demonstrated an inadequate response to the therapy. Subsequently, these patients received treatment with eculizumab. Inclusion criteria were as followed: (1) Confirmed diagnosis of generalized myasthenia gravis; (2) Seropositivity for anti-AChR antibody; (3) Poor early response to efgartigimod; (4) Received at least four infusion of eculizumab (900 mg per week) within four consecutive weeks. Exclusion criteria were as followed: (1) Incomplete baseline records; (2) Clinical manifestations aligning with ocular myasthenia gravis or Myasthenia Gravis Foundation of America (MGFA) classification I; (3) Insufficient dosage and duration of efgartigimod treatment; (4) Suboptimal administration of eculizumab treatment, defined as fewer than 4 infusions or 4 non-consecutive infusions; (5) Converted to other treatments include the addition of conventional immunosuppressants, telitacicept, rituximab, or CAR-T therapy. In this study, we defined “poor early response to efgartigimod” as no clinically meaningful improvement (CMI) being achieved within the first cycle of efgartigimod (10 mg/kg per week for 4 weeks), and the CMI was defined as a reduction of ≥2 points in MG-ADL score (15).

### Data collection

Demographic data, such as age, gender, disease duration, thymic status, and previous treatment were extracted from medical records. Of these 18 patients, 9 provided informed consent for serial blood sampling to monitor IgG levels. Patients with AChR antibody-positive MG and thymoma were classified as thymoma-associated myasthenia gravis (TAMG). Among AChR antibody-positive MG patients without thymoma, those with an onset age younger than 50 years were categorized as early-onset MG (EOMG), while those with an onset age of 50 years or older were categorized as late-onset MG (LOMG).

### Eculizumab administration and follow-up

Eculizumab was administered intravenously with an induction dose of 900 mg infused over 25–45 minutes once weekly for four consecutive weeks, followed by a maintenance dose of 1200 mg every two weeks starting at week 5. Depending on disease severity and patient’s economic status, the dosing interval was gradually extended to every three weeks. To avoid potential interactions, a minimum one-week washout period was maintained between the last efgartigimod infusion and the first eculizumab infusion. Efficacy was evaluated weekly in all 18 patients based on MG-ADL score, QMG score, and minimal symptom expression (MSE). Adverse events were recorded through patient interviews. All 18 patients received prophylactic meningococcal vaccination.

### Statistical analysis

Statistical analyses were performed using SPSS (version 26.0) and GraphPad Prism (version 9.2.0). Categorical variables were presented as numbers (percentages) and compared by the chi-squared test. Normally distributed continuous variables were presented as mean ± standard deviation (SD) and compared by unpaired t-test. Non-normally distributed variables were presented as median (interquartile range, IQR) and compared by Mann–Whitney U test. For the comparison of MG-ADL score, QMG score, and dosage of prednisone across different time points, we performed repeated measures analysis of variance (ANOVA) and Greenhouse-Geisser correction for adjustment. *Post hoc* pairwise comparisons were conducted using Bonferroni correction. Changes in MG-ADL score, QMG score, and dosage of prednisone were expressed as mean change with a 95% confidence interval (CI). A complete-case analysis was applied, and any patient missing data at a given time point was excluded. A two-tailed P value < 0.05 was considered statistically significant.

## Results

### Basic clinical characteristic of the study population

**Figure 1:**
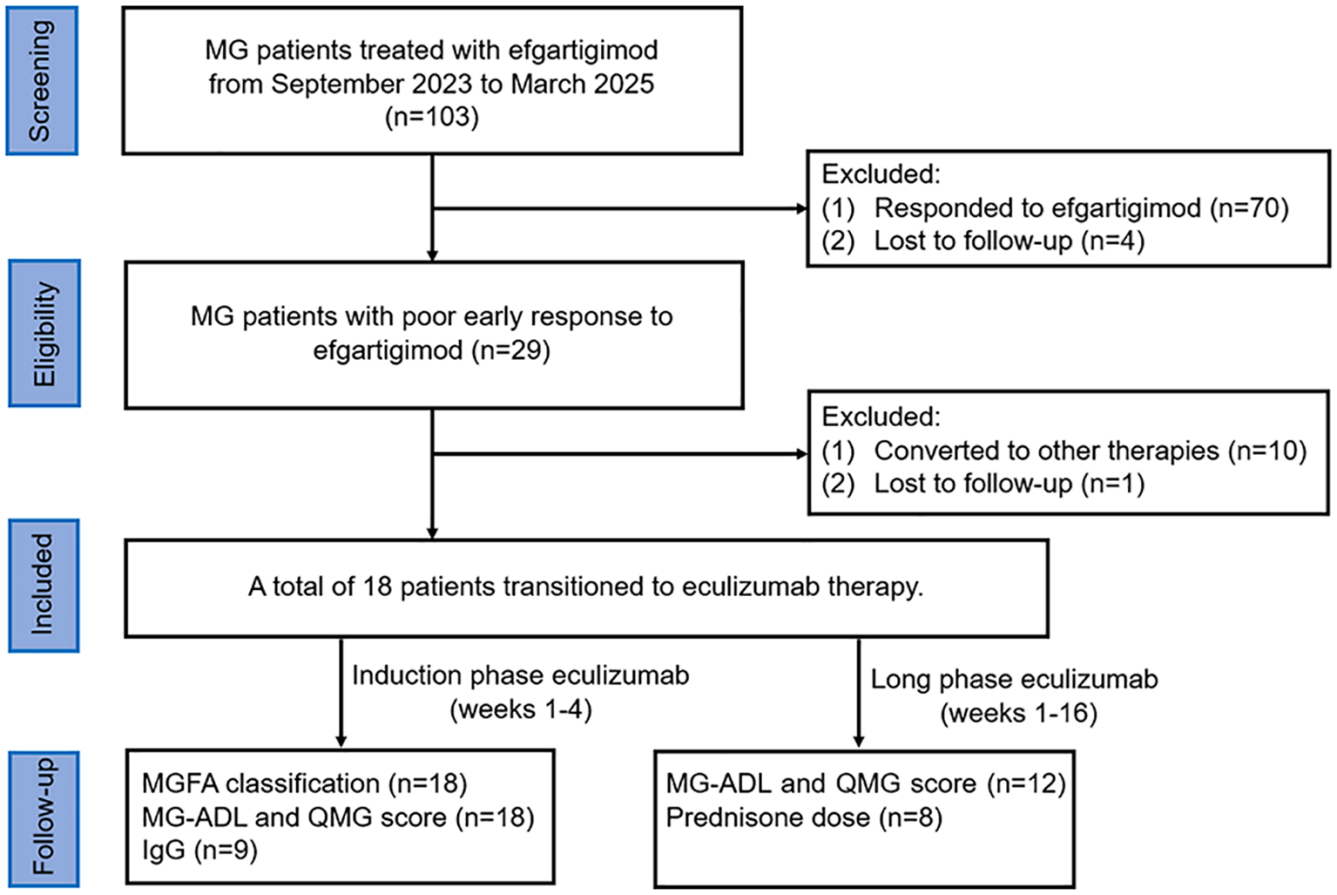
Study flowchart depicting patient enrollment, treatment transitions, and assessment timepoints. MG, myasthenia gravis.

A total of 18 patients who showed a poor early response to efgartigimod and subsequently received treatment with eculizumab were included in the study according to inclusion and exclusion criteria (**Figure 1**). The basic clinical information were presented in **Table 1**. The study population had a mean age of 60.3 ± 15.9 years and mean disease duration of 43.1 ± 45.7 months. The cohort comprised 13 female and 5 male patients. All patients tested positive for anti-AChR antibodies, including six (33.3%) with thymoma. Four patients had previously undergone thymectomy. The mean time interval between the final efgartigimod and the first eculizumab was 28.4 ± 17.0 days. Before efgartigimod, patients were distributed across MGFA classifications as follows: class II (n = 6), class III (n = 9), class IV (n = 2), and class V (n = 1), with a daily dosage of prednisone of 15.0 (10.0, 50.0) mg.

**Table 1:** Baseline characteristics of gMG Patients following poor early response to efgartigimod and switch to eculizumab.

Characteristics	MG (n=18)
Age, years	60.3 ± 15.9
Sex, female, n (%)	13 (73.7)
Age at onset, years	56.7 ± 16.4
Disease duration, months	43.1 ± 45.7
Time between the last efgartigimod and the first eculizumab, days	28.4 ± 17.0
MG subgroup, n (%)	
EOMG	2 (11.1)
LOMG	10 (55.6)
TAMG	6 (33.3)
Thymoma, n (%)	6 (33.3)
Thymectomy, n (%)	4 (22.2)
MGFA classification before efgartigimod, n (%)	
II	6 (33.3)
III	9 (50.0)
IV	2 (11.1)
V	1 (5.6)
MGFA classification before eculizumab, n (%)	
II	6 (33.3)
III	9 (50.0)
IV	1 (5.6)
V	2 (11.1)
Treatments before eculizumab, n (%)	
Pyridostigmine	18 (100.0)
Prednisone	17 (94.4)
Tacrolimus	8 (44.4)
Mycophenolate mofetil	2 (11.1)
Efgartigimod	18 (100.0)
Daily dose of prednisone before efgartigimod, mg	24.2 ± 27.5
Daily dose of prednisone before eculizumab, mg	21.7 ± 24.1
Daily dose of tacrolimus before eculizumab, mg	2.0 ± 0.9
Daily dose of Mycophenolate mofetil before eculizumab, g	1.3 ± 0.4
MG-ADL score before efgartigimod	7.3 ± 3.3
QMG score before efgartigimod	13.9 ± 5.5
MG-ADL score before eculizumab	7.9 ± 3.9
QMG score before eculizumab	14.1 ± 6.6

EOMG, early-onset myasthenia gravis; LOMG, late-onset myasthenia gravis; MG, myasthenia gravis; MG-ADL, myasthenia gravis-specific activities of daily living; MGFA, Myasthenia Gravis Foundation of America; QMG, quantitative myasthenia gravis; TAMG, thymoma-associated myasthenia gravis.

### Inconsistency between decrease in serum IgG and poor early response to efgartigimod

**Figure 2:**
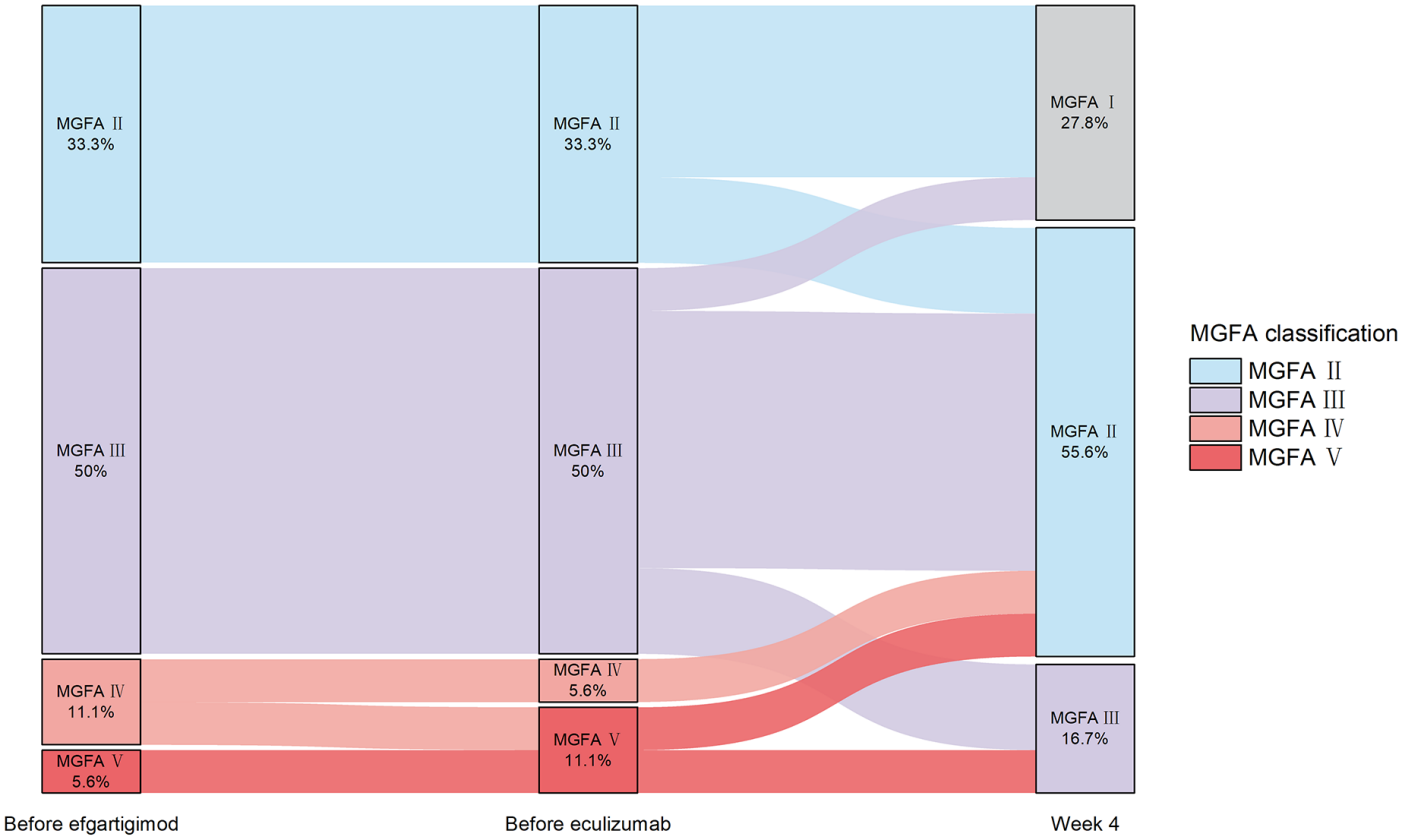
Distribution of MGFA classifications before efgartigimod, before eculizumab, and at week 4 after eculizumab initiation (n=18 at each time point). MGFA, Myasthenia Gravis Foundation of America.

**Figure 3:**
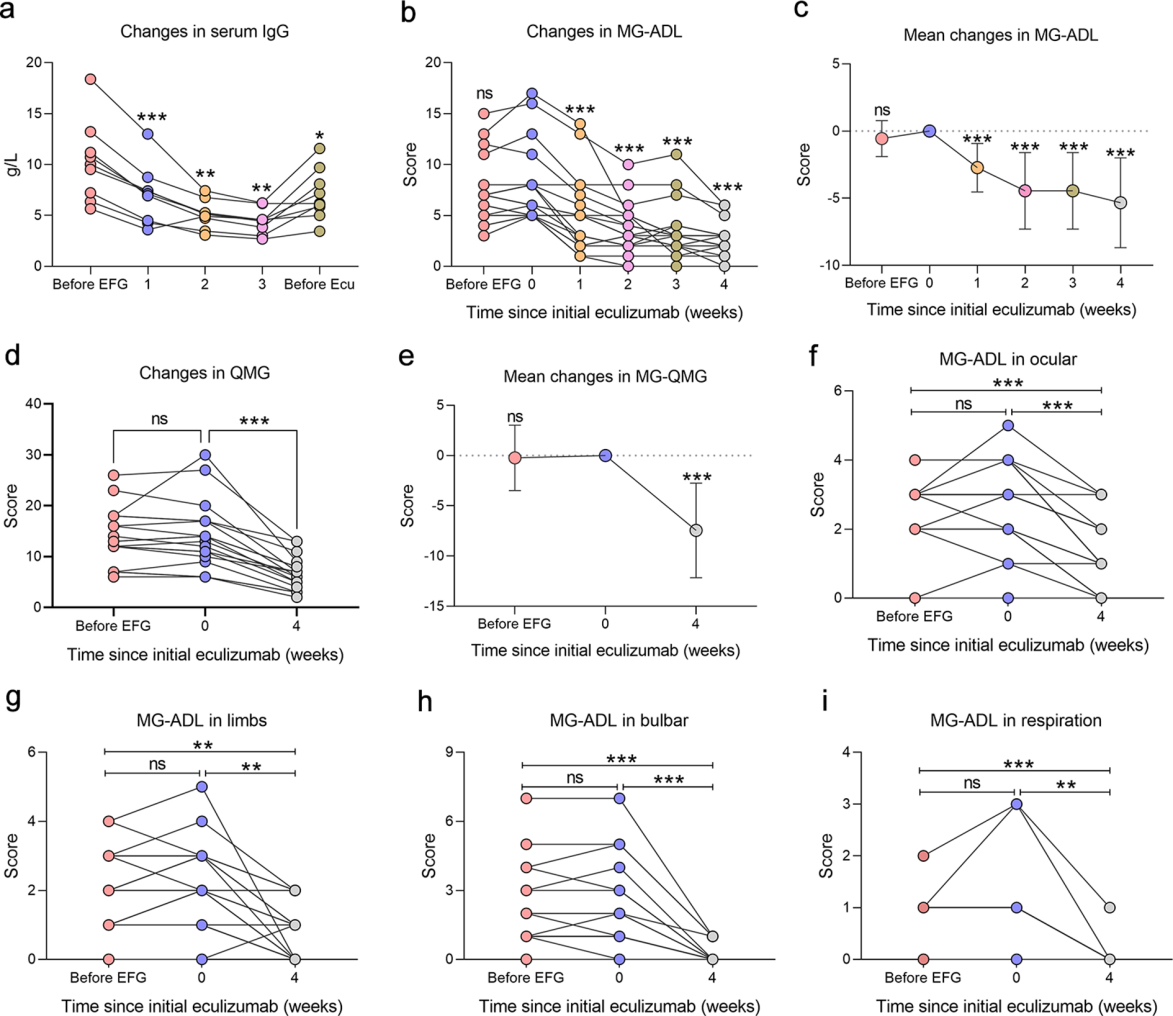
Changes in IgG after efgartigimod and changes in clinical scores before efgartigimod, before eculizumab, and four weeks after eculizumab initiation. **(a)** Changes in serum IgG from before efgartigimod to before eculizumab (n=9 at each time point). **(b–e)** Changes in MG-ADL and QMG scores from before efgartigimod to four weeks after eculizumab initiation (n=18 at each time point). **(f–i)** Changes in MG-ADL score of ocular muscle, limb muscle, bulbar muscle and respiratory muscle (n=18 at each time point). EFG, efgartigimod; IgG, immunoglobulin G; MG-ADL, myasthenia gravis specific activities of daily living; QMG, quantitative myasthenia gravis. *P<0.05, **P<0.01, ***P<0.001, ns:no significance.

The changes in distribution of MGFA classification were shown in **Figure 2**. Following efgartigimod, one patient in MGFA class IV experienced a myasthenic crisis (MGFA class V). Dynamic changes in serum IgG after efgartigimod were measured in nine patients (**Figure 3a**). The baseline level of IgG was 11.2 ± 4.8 g/L. Following treatment with efgartigimod, a significant decrease was observed: -3.3 g/L (95%CI: -4.6 to -2.0) one week after efgartigimod administration; -5.8 g/L (95% CI: -9.1 to -2.5) three weeks after efgartigimod treatment. Even prior to the first eculizumab infusion, the IgG level (7.3 ± 2.5 g/L) was significantly lower than that before efgartigimod treatment (P = 0.020). The decrease in serum IgG did not lead to clinical improvement: MG-ADL and QMG scores were 7.3 ± 3.3 and 13.9 ± 5.5 before efgartigimod, and were 7.9 ± 3.9 and 14.1 ± 6.6, prior to the transition to eculizumab, respectively (**Figures 3b–e**).

### Clinical response to eculizumab within the initial four weeks

All eighteen patients with poor early response to efgartigimod, after receiving at least four infusions of eculizumab, demonstrated marked clinical improvement. Regarding the changes in MGFA classification, two patients initially presenting with myasthenic crisis (MGFA class V) improved to MGFA III and MGFA II, respectively, at week 4 (**Figure 2**). The proportion of patients in MGFA class III decreased from 9/18 (50.0%) to 3/18 (16.7%) by week 4, while the proportion of patients in MGFA class II increased from 6/18 (33.3%) to 10/18 (55.6%) at week 4 (**Figure 2**). Additionally, five patients (27.8%) reached MGFA class I by week 4 (**Figure 2**). The mean MG-ADL score decreased to 5.1 ± 3.7 at week 1, with a reduction of -2.7 (95% CI: -4.0 to -1.5; P < 0.001, **Figures 3b, c**) from day 0; 2.6 ± 1.7 at week 4, with a reduction of -5.3 (95% CI: -7.6 to -3.0; P < 0.001, **Figures 3b, c**). Thirteen patients (72.2%) had CMI with at least a 2-point reduction in the MG-ADL score at week 1, and this number increased to 18 patients (100.0%) at week 4. Besides, four patients (22.2%) attained the minimal symptom expression (MSE, defined as MG-ADL score ≤1) status at week 4. The score in all MG-ADL subdomains improved at week 4: ocular: -1.4 (95% CI: -2.0 to -0.8; P < 0.001, **Figure 3f**); limbs: -1.3 (95% CI: -2.1 to -0.6; P = 0.001, **Figure 3g**); bulbar: -1.9 (95% CI: -2.9 to -0.9; P < 0.001, **Figure 3h**); respiration: -0.7 (95% CI: -1.2 to -0.2; P = 0.003, **Figure 3i**). At week 4, the mean QMG score was 6.5 ± 3.2, showing a significant improvement compared to day 0 [-7.4 (95% CI: -10.2 to -4.7); P < 0.001, **Figures 3d, e**].

### The efficacy of sequential eculizumab treatment

**Figure 4:**
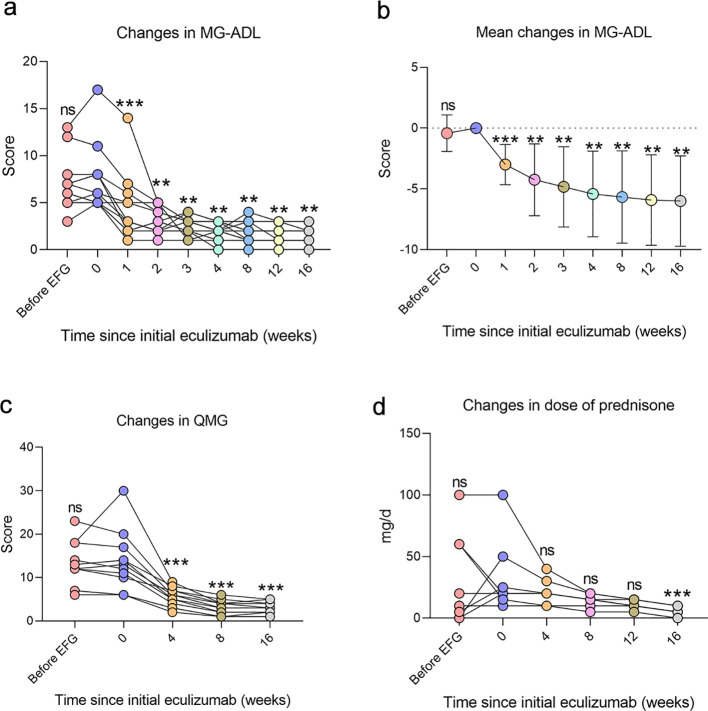
The 16-week efficacy of eculizumab in myasthenia gravis patients with a poor early response to efgartigimod. **(a, b)** Changes in the MG-ADL score within 16 weeks (n=12 at each time point). **(c)** Detailed changes in the QMG score before efgartigimod, and at week 0, 4, 8, 16 since eculizumab initiation (n=12 at each time point). **(d)** Detailed changes in the daily dose of prednisone before efgartigimod, and at week 0, 4, 8, 12, 16 since eculizumab initiation (n=8 at each time point). MG-ADL, myasthenia gravis specific activities of daily living; QMG, quantitative myasthenia gravis. **P<0.01, ***P<0.001, ns:no significance.

Among 18 patients, 12 patients received at least eight times infusion of eculizumab during this study. During 16 weeks of follow-up, all 12 patients (100.0%) showed improvement from week 3 to week 16. The MG-ADL and QMG score of those patients were 7.4 ± 3.6 and 13.2 ± 6.8 before eculizumab. The MG-ADL score decreased to 2.0 ± 1.0 (-5.4 [95% CI: -8.8 to -2.0]; P = 0.002, **Figures 4a, b**) at week 4 and 1.4 ± 0.8 (-6.0 [95% CI: -9.6 to -2.4]; P = 0.001, **Figures 4a, b**) at week 16. The QMG score decreased to 5.4 ± 2.2 [-7.8 (95% CI: -12.3 to 3.3); P = 0.001, **Figure 4c**] at week 4 and 2.9 ± 1.4 (-10.3 [95% CI: -15.7 to -4.9]; P < 0.001, **Figure 4c**) at week 16.

Among the 12 patients, eight patients received prednisone treatment prior to efgartigimod, with a median daily dose of 15.0 (10.0, 60.0) mg. Before eculizumab administration, the number of patients receiving prednisone increased to ten, with a median daily dose of 20.0 (10.0, 31.2) mg (**Figure 4d**). Following treatment with eculizumab, the dosage of glucocorticoids was progressively decreased in tandem with the improvement of symptoms: 15.0 (10.0, 16.2) mg per day at week 8 (P = 0.240, **Figure 4d**); 10.0 (10.0, 15.0) mg per day at week 12 (P = 0.050, **Figure 4d**); 5.0 (5.0, 10.0) mg per day at week 16 (P < 0.001, **Figure 4d**). The daily dose of prednisone for seven patients was less than or equal to 5 mg per day at week 16.

**Figure 5:**
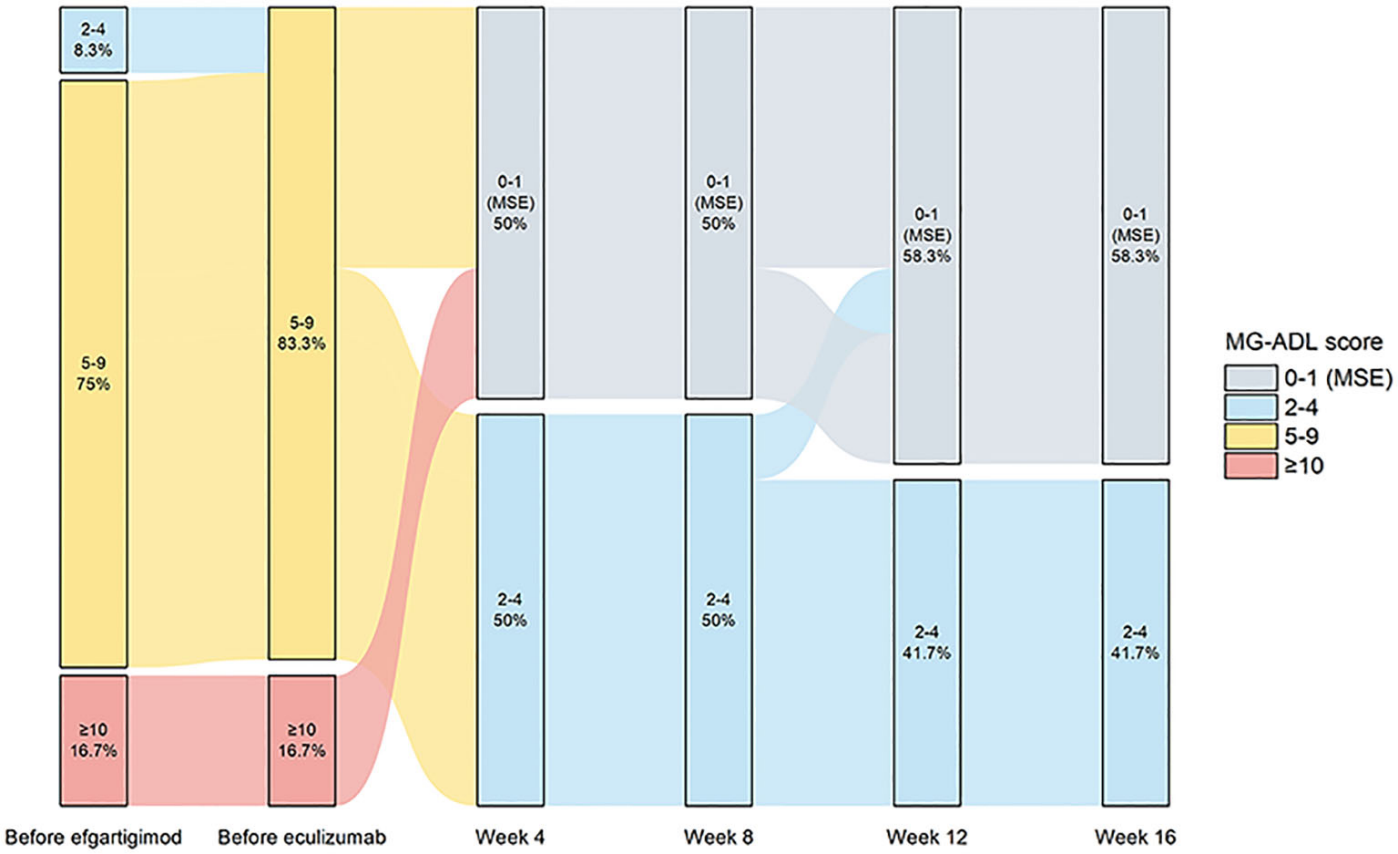
The proportion of patients with different MG-ADL score before efgartigimod, and at week 0, 4, 8, 12, 16 since eculizumab initiation (n=12 at each time point). MG-ADL, myasthenia gravis specific activities of daily living; MSE, minimal symptom expression.

Stratifying the severity of MG patients based on the MG-ADL score: the proportion of patients with an MG-ADL score ranging from 5 to 9 decreased from 10/12 (83.3%) prior to eculizumab to 0 starting from week 4; the proportion of patients with an MG-ADL score of 2 to 4 increased to 6/12 (50.0%) from 0 at week 4.; 6/12 patients (50.0%) attained the MSE at week 4, and this proportion increased to 7/12 (58.3%) at week 12 (**Figure 5**).

### The safety of sequential eculizumab treatment

Most treatment-emergent adverse events (AEs) were mild in intensity. No patients experienced serious AEs, including myasthenia gravis crisis or disease worsening. The most commonly reported AEs were headache [6 patients (33.3%)] and nasopharyngitis [4 patients (22.2%)]. Mild lower urinary tract infection and lower limb edema each occurred in one patient. Notably, none of the following commonly monitored AEs were observed: vomiting, extremity pain, pyrexia, abdominal pain, diarrhea, decreased appetite, palpitations, upper respiratory tract infection, or lymphopenia. Additionally, there were no deaths, meningococcal infections, clinically meaningful findings with regard to abnormal laboratory results (**Supplementary Figure 1**), electrocardiogram findings, physical examination, and vital signs.

## Discussion

This real-world cohort study suggested that eculizumab could bring about rapid and clinically significant improvement in patients with AChR antibody-positive gMG who exhibited a poor early response to efgartigimod. In this study, an interesting dissociation was observed between the reduction of IgG and the clinical response. Specifically, although efgartigimod led to substantial decreases in serum IgG levels, this did not necessarily result in clinical improvement. Indeed, the correlation between the titers of AChR antibody and disease severity remains limited (16–18). It is possible that some patients may have ongoing complement-mediated pathology despite reductions in circulating IgG (19), although biomarkers were not assessed in this study. A real-world study indicated that 9 TAMG patients who did not respond to efgartigimod showed significant improvement after switching to eculizumab (20). Complement inhibitors may exert unique therapeutic effects in patients with suboptimal responses to efgartigimod, as evidenced by the rapid clinical improvement following eculizumab administration (21). In our cohort, the sustained benefits over 16 weeks of follow-up, during which over half of the patients achieved MSE and were able to reduce prednisone doses to 5 mg or less per day, further strengthens the case for complement inhibition as an effective sequential therapy when FcRn modulation proves ineffective. However, corticosteroid dosages were adjusted upward based on clinical assessment between efgartigimod and eculizumab use in certain cases. These concomitant adjustments in immunosuppressive therapy constitute important confounding factors to consider. Therefore, the clinical improvements observed after switching to eculizumab might partly reflect steroid effects rather than eculizumab alone. Furthermore, our study found that IgG levels prior to eculizumab infusion remained lower than their pre-efgartigimod treatment baseline, suggesting that the immunomodulatory effects of efgartigimod may have persisted. This overlap must be considered a key factor when interpreting the subsequent therapeutic response to eculizumab.

When compared with the REGAIN study (22), the magnitude and speed of improvement with eculizumab appeared more favorable, a difference that may be partially explained by the early administration of efgartigimod to inhibit pathogenic antibodies upstream, thereby enhancing the efficacy of complement inhibition. Particularly, two patients presenting with myasthenic crisis, showed rapid improvement to MGFA class II/III within 4 weeks. This may provide some evidence for the treatment of myasthenic crisis with eculizumab. In fact, several case reports and small-sample cohort studies have found that eculizumab has the potential to be used as an emergency treatment for myasthenic crisis (23–26). Efgartigimod, as a novel biological agent, has also been reported to be used as a salvage treatment for patients with myasthenic crisis or impending crisis, and even has a better effect than traditional intravenous immunoglobulin (27, 28). However, head-to-head clinical trials are still required to determine the optimal drug selection between these two agents during exacerbations and crises. In addition, other complement C5 inhibitors such as ravulizumab and zilucoplan are emerging, and these complement inhibitors did not show significant differences in terms of clinical efficacy and safety, representing alternative therapies for gMG patients with poor early responses to efgartigimod (29). Another concern is the infection risk associated with the emergency use of complement inhibitors, particularly Neisseria meningitidis infections. The safety profile observed in our cohort was reassuring, with only two mild adverse events reported. An important practical advantage is that eculizumab does not induce hypogammaglobulinemia, making it particularly suitable for administration following therapies such as efgartigimod, intravenous immunoglobulin, or plasma exchange, which significantly reduce IgG levels. The dosing flexibility demonstrated in our study, even with extended intervals, allowed a subset of patients to successfully transition to a once-every-three-week maintenance regimen without compromising therapeutic efficacy, potentially contributing to reduced economic burden associated with long-term treatment.

This study has several limitations. First, current research reports, including our study, indicate that 20% to 30% of patients with gMG exhibit suboptimal responses to efgartigimod treatment. Although we prospectively enrolled 103 gMG patients receiving efgartigimod and conducted rigorous follow-up and assessment, the growing use and demonstrated efficacy of FcRn antagonists have influenced patient availability, thereby limiting the sample size and generalizability of this study. To address these limitations, future multicenter studies are planned to expand the cohort and further validate these findings. Second, the absence of a control group precludes direct comparative analysis with other therapeutic approaches. Third, no further investigation into potential biomarkers (e.g., AChR-Ab levels, CH50, C5b-9, C3/C4 or IgG subclasses) or underlying mechanisms was undertaken in patients with suboptimal response to efgartigimod. Fourth, the current cohort may include patients who simply had not yet reached maximal response to FcRn inhibition. Some patients may require more treatment cycles of efgartigimod to achieve a response. However, in this study, patients with suboptimal improvement after one treatment cycle opted for alternative therapies (such as complement inhibitors) due to physiological and psychological factors, which may lead to an underestimation of efgartigimod responders. Moreover, IgG levels remained lower than the baseline before the initiation of eculizumab. This temporal overlap between residual efgartigimod effect and early eculizumab exposure substantially confounds attribution of clinical benefit. Fifth, our study specifically focused on the patients who had an suboptimal early response to efgartigimod, the cohort is highly selected and at risk for biases such as confounding by disease severity or treatment resistance.

## Conclusion

In conclusion, this real-world study provides preliminary evidence that eculizumab demonstrates rapid and significant efficacy, along with a favorable safety profile, in patients with gMG who exhibit a poor early response to efgartigimod. These findings suggest that complement inhibition may serve as a potential sequential therapeutic strategy after early failure of FcRn antagonist therapy.

## Data Availability

The raw data supporting the conclusions of this article will be made available by the authors, without undue reservation.
