# Supplementary material for: Real-world efficacy of eculizumab in generalized myasthenia gravis patients with poor early response to efgartigimod: a prospective cohort study
Source: Front Immunol. 2026 May 25;17:1730742. doi: 10.3389/fimmu.2026.1730742 (PMC13243283; doi:10.3389/fimmu.2026.1730742)
Supplement: Supplementary file 1 [file Table1.docx]

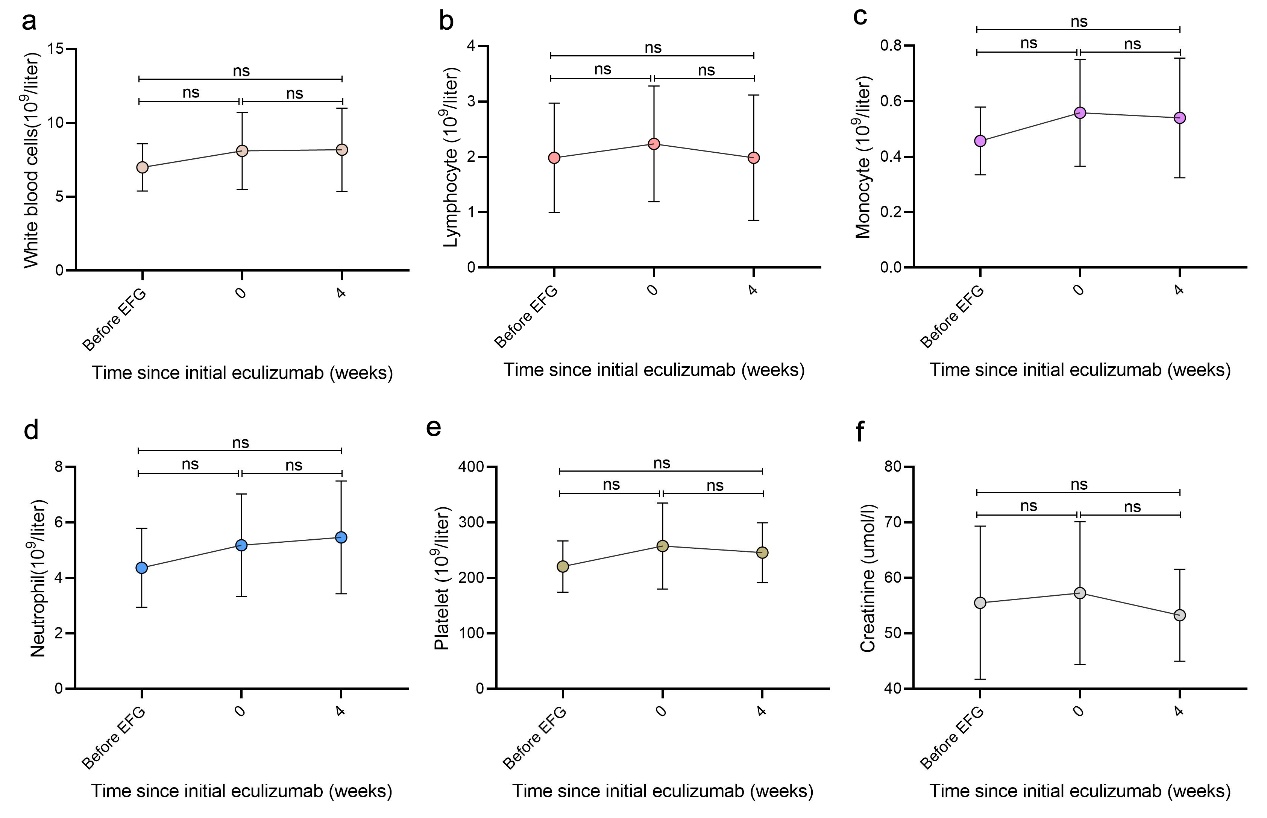


Supplementary Figure 1. Changes in white blood cells, lymphocytes, monocytes, neutrophil, platelet and creatinine before efgartigimod, before eculizumab, and four weeks after eculizumab initiation (n=12 at each time point).

**Supplementary table 1. Baseline clinical characteristics of gMG patients with poor early response to efgartigimod**

| Characteristics | Included MG (n=18) | Excluded MG (n=11) | P |
| --- | --- | --- | --- |
| Age, years | 60.3±15.9 | 49.27±16.86 | 0.087 |
| Sex, female, n (%) | 13 (73.7) | 6(63.6) | 0.628 |
| Age at onset, years | 56.7±16.4 | 46.00±18.33 | 0.108 |
| Disease duration, months | 43.1±45.7 | 40.8±53.9 | 0.906 |
| MG subgroup, n (%) |  |  | 0.455 |
| EOMG | 2 (11.1) | 3(27.2) |  |
| LOMG | 10 (55.6) | 4(36.4) |  |
| TAMG | 6 (33.3) | 4(36.4) |  |
| Thymoma, n (%) | 6 (33.3) | 4(36.4) | 0.868 |
| Thymectomy, n (%) | 4 (22.2) | 4(36.4) | 0.408 |
| MGFA classification before efgartigimod, n (%) |  |  | 0.809 |
| Ⅱ | 6 (33.3) | 3(27.3) |  |
| Ⅲ | 9 (50.0) | 6(54.5) |  |
| Ⅳ | 2 (11.1) | 2(18.2) |  |
| Ⅴ | 1 (5.6) | 0(0.0) |  |
| Treatments before efgartigimod, n (%) |  |  |  |
| Pyridostigmine | 18 (100.0) | 11(100.0) |  |
| Prednisone | 17 (94.4) | 11(100.0) |  |
| Tacrolimus | 8 (44.4) | 4(36.4) |  |
| Mycophenolate mofetil | 2 (11.1) | 0(0.0) |  |
| MG-ADL score before efgartigimod | 7.3±3.3 | 7.0±1.5 | 0.757 |
| QMG score before efgartigimod | 13.9±5.5 | 11.7±4.3 | 0.275 |

Abbreviation: EOMG, early-onset myasthenia gravis; LOMG, late-onset myasthenia gravis; MG, myasthenia gravis; MG-ADL, myasthenia gravis-specific activities of daily living; MGFA, Myasthenia Gravis Foundation of America; QMG, quantitative myasthenia gravis; TMG, thymoma-associated myasthenia gravis.

**Supplementary table 2. Prednisone adjustments in the 18 patients during the periods preceding efgartigimod initiation and eculizumab initiation**

| Patients | Prednisone, mg | |
| --- | --- | --- |
|  | before efgartigimod | before  eculizumab |
| P1 | 100 | 100 |
| P2 | 5 | 5 |
| P3 | 10 | 10 |
| P4 | 0 | 20 |
| P5 | 10 | 10 |
| P6 | 15 | 15 |
| P7 | 10 | 10 |
| P8 | 60 | 20 |
| P9 | 5 | 25 |
| P10 | 30 | 5 |
| P11 | 50 | 40 |
| P12 | 0 | 0 |
| P13 | 60 | 50 |
| P14 | 0 | 0 |
| P15 | 10 | 10 |
| P16 | 20 | 20 |
| P17 | 40 | 40 |
| P18 | 10 | 10 |
